# Impact of Endovascular Aortic Repair on Aortic Stiffness: Preliminary Results from a Prospective In Vivo Study Following EVAR

**DOI:** 10.3390/jcm15041532

**Published:** 2026-02-15

**Authors:** Paolo Bellotti, Emma-Lena Maris, Jasper F. de Kort, Daniele Bissacco, Silvia Romagnoli, Maurizio Domanin, Chiara Grignaffini, Paolo Salvi, Gianfranco Parati, Valentina Mazzi, Karol Calo, Bianca Griffo, Diego Gallo, Umberto Morbiducci, Constantijn E. V. B. Hazenberg, Joost A. van Herwaarden, Santi Trimarchi

**Affiliations:** 1 Department of Vascular Surgery, Fondazione IRCCS Ca’ Granda Ospedale Maggiore Policlinico, 20122 Milan, Italy; paolo.bellotti@policlinico.mi.it (P.B.); jasperdkort@gmail.com (J.F.d.K.); danielebissaccomd@gmail.com (D.B.); silvia.romagnoli@policlinico.mi.it (S.R.); maurizio.domanin@unimi.it (M.D.); chiara.grignaffini@unimi.it (C.G.); santi.trimarchi@unimi.it (S.T.); 2 Department of Vascular Surgery, University Medical Center Utrecht, 3584 Utrecht, The Netherlands; c.e.v.b.hazenberg@umcutrecht.nl (C.E.V.B.H.); j.a.vanherwaarden@umcutrecht.nl (J.A.v.H.); 3 Department of Clinical and Community Sciences, University of Milan, 20157 Milan, Italy; 4 Independent Researcher, 20100 Milan, Italy; psalvi.md@gmail.com; 5 Department of Cardiovascular, Neural and Metabolic Sciences, Italiano Auxologic Institute IRCCS, 20145 Milan, Italy; gianfranco.parati@unimi.it; 6 Department of Medicine and Surgery, University of Milano-Bicocca, 20126 Milan, Italy; 7 Department of Mechanical and Aerospace Engineering, Politecnico di Torino, 10129 Torino, Italy; valentina.mazzi@polito.it (V.M.); karol.calo@polito.it (K.C.); bianca.griffo@polito.it (B.G.); diego.gallo@polito.it (D.G.); umberto.morbiducci@polito.it (U.M.)

**Keywords:** Endovascular Procedures, Endovascular Aortic Repair, Vascular Surgery, Vascular Stiffness, Arterial Hemodynamics, Pulse Wave Velocity, Cardiovascular Diseases

## Abstract

**Background**: Aortic stiffness (AoS) is an established predictor of cardiovascular morbidity and mortality. Endovascular aneurysm repair (EVAR) introduces a rigid stent-graft into the aorta, potentially increasing AoS and impairing subendocardial perfusion. This prospective study aimed to evaluate changes in AoS and myocardial perfusion following EVAR, measured by carotid-to-femoral pulse wave velocity (cf-PWV) and the Subendocardial Viability Ratio (SEVR), and examined the influence of graft length on post-operative cf-PWV and SEVR. **Methods**: From October 2023 to April 2025, 38 patients undergoing elective EVAR were prospectively enrolled. Cf-PWV and the SEVR were measured <72 h preoperatively and 7 days postoperatively using the PulsePen^®^ device. Descriptive statistics were used to summarize baseline characteristics. Data were assessed for normality with the Shapiro–Wilk test; non-normally distributed variables were analysed using the Wilcoxon signed-rank test and presented as median [interquartile range, IQR], while normally distributed variables were analysed using paired t-tests and presented as mean ± standard deviation (SD). Linear regression was applied to evaluate associations between graft length and postoperative changes in cf-PWV and SEVR. **Results**: Cf-PWV increased significantly after EVAR, with a median within-patient change of 1.0 m/s [IQR 3.1] (*p* < 0.001), corresponding to a 10.6% increase. The SEVR decreased significantly by 15.1% (*p* = 0.006). Graft length correlated positively with cf-PWV change, with a 0.2% increase in cf-PWV per millimetre of graft length (r = 0.41; *p* = 0.029), but not with SEVR (r = 0.058, *p* = 0.763). **Conclusions**: EVAR was associated with increased AoS and reduced subendocardial perfusion, with greater stiffness changes observed in patients receiving longer grafts. These preliminary findings highlight important haemodynamic consequences of EVAR and may inform patient selection, postoperative management, and the development of future stent-graft designs to mitigate long-term cardiovascular risk.

## 1. Introduction

Aortic stiffness (AoS) is increasingly recognized as a major predictor of cardiovascular morbidity and mortality [1]. Aortic stiffness refers to the loss of elasticity and distensibility of the aortic wall, resulting in reduced capacity of the vessel to expand during systole and recoil during diastole, a phenomenon known as the Windkessel effect [2].

Under normal physiological conditions, the aorta buffers the pulsatile flow generated by the left ventricle (LV), maintaining continuous blood flow during diastole [3]. Increased AoS impairs this buffering function of the aorta, leading to elevated systolic pressure, reduced diastolic pressure, and widened pulse pressure [3]. These haemodynamic alterations increase left ventricular afterload, reduce coronary perfusion, and enhance pulsatile energy transmission to microvascular beds, particularly in the brain and kidneys [3,4,5]. These haemodynamic changes caused by AoS may promote LV hypertrophy, myocardial fibrosis and dysfunction, and cerebral and renal blood flow impairment [3,6].

AoS is commonly quantified by pulse wave velocity (PWV), which reflects the speed of pulse wave propagation along the aortic wall following left ventricular ejection [3,7]. An increase in AoS results in a faster wave propagation along the vascular system, indicated by an increase in PWV. The Subendocardial Viability Ratio (SEVR), also known as the Buckberg index, provides complementary information by estimating the balance between myocardial oxygen supply and demand, serving as a parameter for myocardial perfusion [3]. PWV and SEVR can therefore assess the mechanical and functional consequences of arterial stiffening.

While AoS is influenced by aging, hypertension, and vascular disease, recent evidence suggests that endovascular aortic repair may also affect aortic compliance [8,9,10,11]. Both clinical and ex vivo studies have demonstrated that EVAR can increase PWV, even relative to open surgical repair (OSR) [12,13]. Current guidelines recommend endovascular procedures as the preferred treatment for abdominal (EVAR) aortic pathologies due to their minimally invasive nature, reduced perioperative morbidity, and shorter recovery [14,15,16]. However, the implantation of a rigid stent-graft can alter the viscoelastic properties of the native aorta, increasing PWV and potentially elevating cardiovascular risk [9].

Given the cardiovascular consequences of AoS and the growing use of endovascular repair, it is essential to understand the cardiovascular implications of stent-graft implantation. While ex vivo and animal studies have demonstrated increases in PWV following stent-graft implantation, prospective in vivo data in patients undergoing EVAR remain limited [10,17,18].

This article reports the preliminary descriptive findings of an ongoing prospective study aiming to evaluate changes in aortic stiffness and myocardial perfusion in patients undergoing EVAR, as measured by PWV and SEVR. A secondary objective of this study is to evaluate the relationship between graft length and postoperative changes in PWV and SEVR, thereby clarifying its potential role in aortic stiffness. Based on previous literature suggesting that stent-graft implantation increases aortic stiffness, the primary hypothesis of this study is that EVAR leads to an increase in PWV and a corresponding decrease in SEVR, reflecting impaired myocardial perfusion. The secondary hypothesis is that the extent of these changes may be influenced by graft length, with longer stent-grafts associated with greater alterations in PWV and SEVR.

## 2. Methods

A detailed protocol for this study was previously published in a peer-reviewed journal at the start of patient recruitment [19]. From 1 October 2023 to 30 April 2025, a prospective study was conducted, including patients scheduled for endovascular aortic repair at Fondazione IRCCS Ca’ Granda Ospedale Maggiore Policlinico in Milan, Italy. This analysis is part of this ongoing prospective study investigating the effects of EVAR and TEVAR on aortic stiffness and pulse wave velocity; the present manuscript reports results for the EVAR cohort only. The study comprised three phases: initial recruitment and collection of baseline patient data before surgery, preoperative assessment, and postoperative evaluation.

### 2.1. Patient Selection

The study was conducted in accordance with the Declaration of Helsinki and was approved by the institutional ethics committee on 27 June 2025 (ID 6170). Written informed consent was obtained from all participants before their inclusion in the study.

Patients were eligible if they were adults (≥18 years) undergoing elective EVAR for aortic aneurysms using any type of commercially available endograft (standard, fenestrated, branched, or custom-made) and an anticipated survival of more than one year.

Exclusion criteria included emergency endograft implantation, pregnancy or lactation, obesity (BMI > 40 kg/m^2^), cardiac instability, persistent atrial fibrillation, valvular heart disease, history of bilateral carotid endarterectomy and/or carotid stenting, significant stenosis (>70%) of the carotid or femoral arteries, skin integrity issues in the neck and/or groin precluding measurements, or concomitant surgical interventions.

### 2.2. Data Collection

Demographic characteristics, comorbidities, smoking status, and medication use were retrieved from medical records. Comorbidities that were documented included hypertension, dyslipidaemia, diabetes mellitus, atrial fibrillation, chronic kidney disease (CKD), chronic obstructive pulmonary disease (COPD), a history of coronary artery bypass grafting (CABG), and the presence of a pacemaker. Medication data included the use of antihypertensives, beta-blockers, alpha-blockers, antiarrhythmics, antiplatelet and anticoagulant agents, statins, and proton pump inhibitors.

Vital parameters at admission were recorded, including heart rate, oxygen saturation (SpO_2_), and body temperature. In addition, laboratory values were measured, including red blood cell count, haematocrit, haemoglobin concentration, blood glucose level, and creatinine levels.

### 2.3. Measurements and Data Acquisition

Patients were clinically evaluated through vascular evaluation, including assessment of peripheral pulses, signs of peripheral ischemia or venous disease, and manifestations of atherosclerosis. The diagnostic work-up and the indication for EVAR were established in accordance with recent guidelines [20,21]. The endovascular procedure was performed without modifications to the standard intra- and postoperative protocol, and all interventions were performed by an experienced vascular surgeon (>30 aortic procedures per year). EVAR devices were selected following standard clinical practice, based on patient anatomy, device availability, and manufacturer instructions for use, without study-driven selection. The following devices were used: W. L. Gore & Associates, Inc. (Flagstaff, AZ, USA); Medtronic, Inc. (Minneapolis, MN, USA); Terumo Corporation (Tokyo, Japan); Endologix LLC (3910 Brickway Blvd, Santa Rosa, CA, USA); and Cook Medical Europe Ltd. (Limerick, Ireland).

Once enrolled, AoS was assessed by measuring the PWV using the PulsePen^®^ device (DiaTecne, San Donato Milanese, Italy) within 72 h before and 7 days after intervention. Preoperative measurements within 72 h prior to surgery were chosen to provide a stable baseline under controlled conditions prior to interventions, while postoperative assessment at 7 days was intended to capture early, acute haemodynamic changes following stent-graft implantation. Patients abstained from caffeine and alcohol for at least 12 h before measurement.

**Figure 1:**
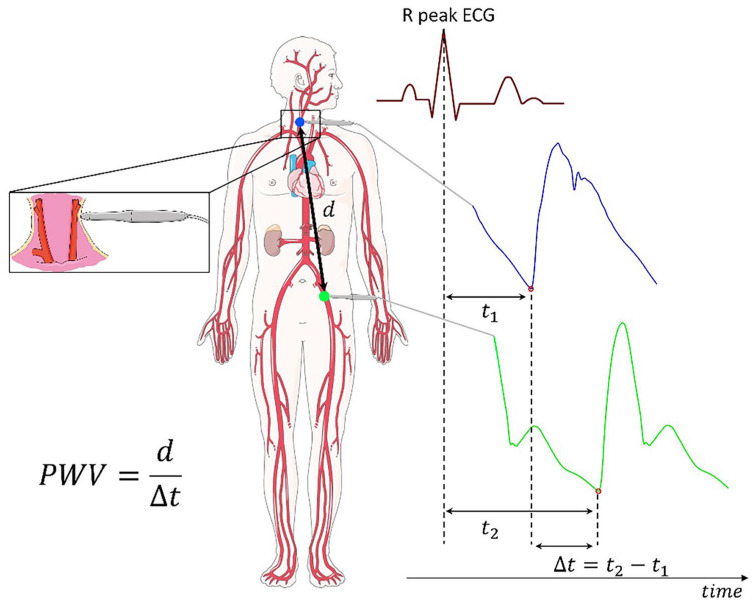
Schematic illustration of pulse wave velocity (PWV) assessment through applanation tonometry. Carotid (blue line) and femoral (green line) pressure waves are aligned to the R peak of the QRS complex of the ECG signal to determine the transit time (Δ*t*) of the pressure wave. Distance (d) is calculated by multiplying the distance between the carotid and the femoral artery by 0.8. PWV is calculated by dividing the d by Δ*t*. Reprinted with permission from Minerva Cardiology and Angiology [19].

The applanation tonometry device is a pocket-sized, wireless system composed of a receiver, a tonometer, and an electrocardiogram (ECG) unit. The blood pressure waveform is calibrated on the values of diastolic blood pressure and mean arterial pressure measured in the brachial artery using a validated sphygmomanometer. Blood pressure measurements were performed simultaneously with the blood pressure waveform recording using tonometry. For accurate readings, the tonometer was positioned perpendicular to the skin at the site of maximal arterial pulsation. Gentle pressure was applied to flatten the arterial wall, enabling the sensor to record pressure waveforms corresponding to the intra-arterial pressure at the vessel’s centre. Cf-PWV was measured at rest at the carotid and femoral sites, with three repeated recordings obtained at each site. The mean pulse graph obtained from the measurements was used for analysis. The timing of each measurement was referenced to the R wave of the ECG QRS complex. Waveform quality was continuously assessed during acquisition through real-time software feedback and only recordings meeting the predefined quality criteria were stored. For analysis, the waveforms were subsequently reviewed and the highest-quality traces were selected by an experienced investigator (PS). To measure the carotid-to-femoral distance, the distance between the carotid and femoral sites was measured directly and multiplied by 0.8 for analysis according to the European Society of Cardiology (ESC) guideline [22]. PWV was calculated according to formulas and recommendations previously published by Salvi et al. [3]. According to the ESC guidelines, an indirectly measured PWV of 9.6 m/s and a directly measured PWV of 12 m/s are considered cut-off values, above which individuals are at increased risk of cardiovascular morbidity and complications [22]. A schematic overview of the PWV measurement is shown in Figure 1.

The PulsePen^®^ is validated as a non-invasive portable tonometer for measuring arterial PWV, demonstrating no significant differences compared to simultaneous invasive and tonometry reference measurements, with acceptable inter- and intra-observer reproducibility [23]. Pulse wave acquisition with the PulsePen^®^ device requires careful probe placement and maintaining a stable contact with the artery. The device is sensitive to even small movements, and obtaining an optimal waveform can be challenging, particularly in patients who are unable to lie fully supine or remain completely still. The PulsePen^®^ software (version 2.3.3) analyses individual pulse waveforms and calibrates them to mean and diastolic blood pressure, which minimizes the impact of minor respiratory or movement-related fluctuations on the calculated PWV. Nevertheless, in the postoperative EVAR setting, several recordings did not meet quality criteria due to practical constraints such as limited patient mobility, presence of catheters, or suboptimal operator posture.

The SEVR is a pulse-wave derived parameter that assesses the balance between myocardial oxygen supply and demand. It is calculated from parameters derived from the morphology of the aortic pressure wave and from the measurement of the pulse wave velocity [3]. A decrease in SEVR reflects a worsening oxygen balance, where myocardial oxygen demand exceeds supply, indicating potential myocardial ischemia. Various cut-off values for low SEVR have been reported in the literature, ranging from below 150% in studies identifying coronary microvascular dysfunction to below 90% in other cohorts as a predictor of cardiovascular mortality [24,25]. SEVR values less than 45% have been shown to relate to ischemic subendocardial damage due to a mismatch between oxygen supply and consumption [3].

For the analysis examining the relationship between graft length and changes in cf-PWV and SEVR, graft length was defined as the nominal length of the endograft main body; iliac limb extensions were excluded from this measurement. Graft length was determined based on the device characteristics during preoperative planning and confirmed by manufacturer specifications for the implanted endografts, as recorded in the procedural documentation. This information reflects the nominal implanted graft length.

### 2.4. Study Outcomes

The primary outcome of the study was the change in cf-PWV before and after EVAR.

Secondary outcomes included postoperative changes in the SEVR after EVAR procedures. Furthermore, the relationship between total graft length and postoperative changes in cf-PWV and SEVR was evaluated.

### 2.5. Statistical Analysis

Descriptive statistics were used to summarize baseline characteristics, which are presented as means ± standard deviations (SD) for continuous data and counts with percentages for categorical data.

Haemodynamic parameters (cf-PWV and SEVR) were assessed for normality using the Shapiro–Wilk test. For variables that were not normally distributed, values were presented as median [interquartile range (IQR)], and pre- and post-EVAR differences were analysed using the Wilcoxon signed-rank test. In addition, the median within-patient change was calculated by first computing the difference between post- and pre-EVAR values for each patient and then taking the median of these differences. For descriptive purposes, percentage change per patient was also computed as (Post-Pre)/Pre × 100. Variables that were normally distributed were presented as mean ± standard deviation (SD), and pre- and post-EVAR differences were analysed using a paired *t*-test. Linear regression analysis was applied to evaluate the relationship between graft length and postoperative changes in cf-PWV and the SEVR, with Pearson’s correlation coefficient (r) used to quantify the strength of the association. Statistical significance was defined as *p* < 0.05. All analyses were performed in R (version 5.5) using the ggplot2 package (version 3.5.2).

## 3. Results

**Figure 2:**
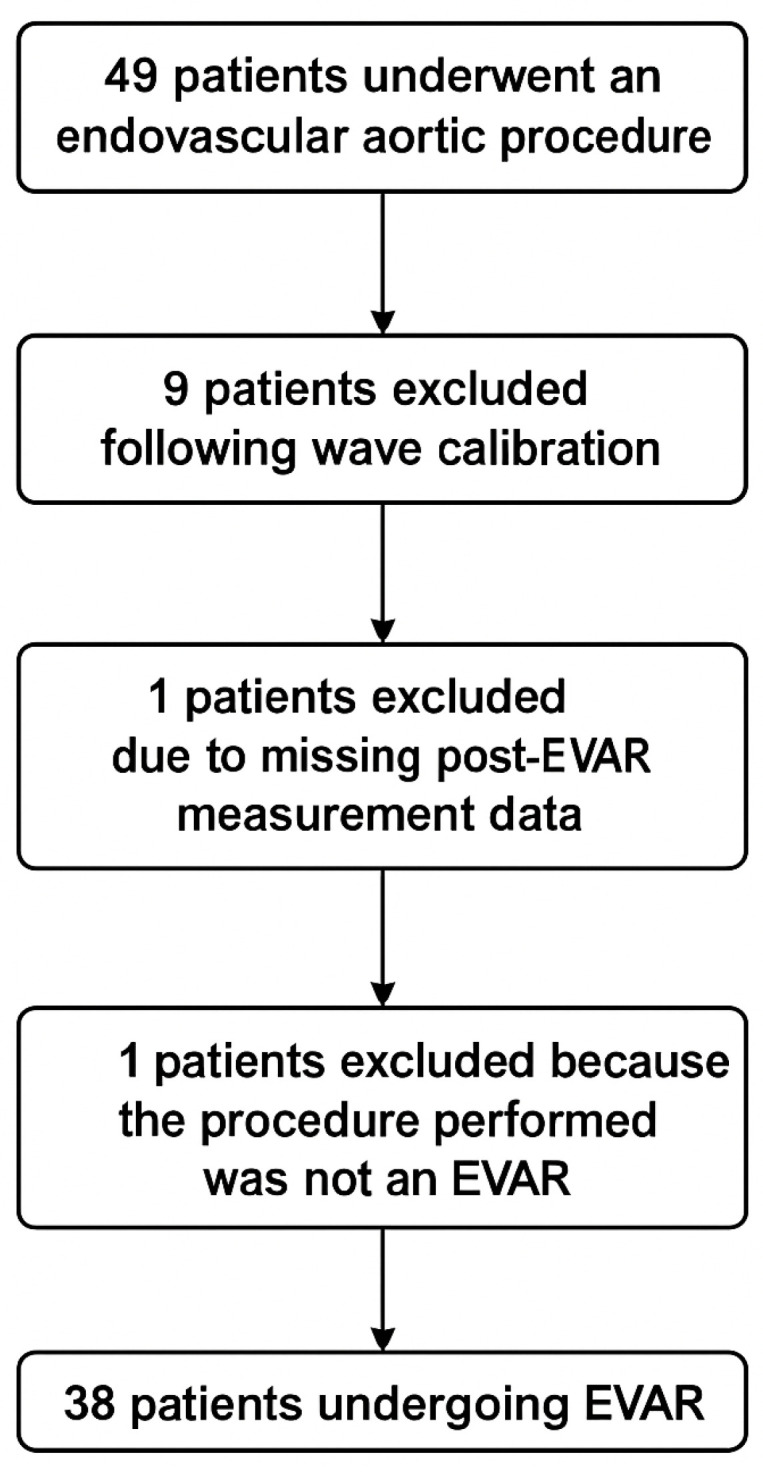
Flow diagram of included patients.

During the study period, 49 patients underwent an endovascular aortic procedure. Of these, 9 were excluded following wave calibration. One patient was excluded due to missing post-EVAR measurement data, and one patient was excluded because of conversion to an open procedure (Figure 2). The final study cohort consisted of 38 patients undergoing EVAR. Demographic characteristics are summarized in Table 1, while perioperative findings are presented in Table 2. The cohort was predominantly male (89%) and mostly aged ≥65 years (82%). Key comorbidities included hypertension (63%) and dyslipidaemia (39%), with lower prevalence of diabetes (13%), atrial fibrillation (18%), CKD (3%), and COPD (8%). Over half of the patients were on antihypertensive therapy (53%) and statins (53%), reflecting the high cardiovascular risk profile of the population. Baseline vital signs and laboratory values were within normal ranges.

### 3.1. Pulse Wave Velocity

**Figure 3:**
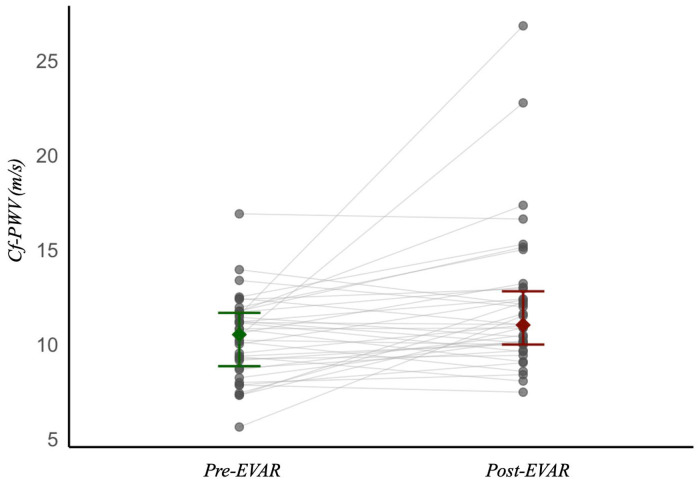
Cf-PWV pre- and post-EVAR (10.6% increase, *p* < 0.001). Each dot represents an individual patient measurement, and lines connect paired pre-EVAR and post-EVAR measurements within the same patient.

PWV data were not normally distributed. PWV increased significantly following EVAR, from 10.51 [IQR 2.82] m/s preoperatively to 11.02 [IQR 2.81] m/s postoperatively (*p* < 0.001), with a median within-patient increase of 1.0 [IQR 3.1] m/s, corresponding to a 10.6% [IQR 28.8%] rise. Median cf-PWV changes did not differ significantly between prosthesis types (*p* = 0.42). Figure 3 shows the changes in cf-PWV pre- and postoperatively, demonstrating an overall upward shift post-EVAR.

Of the 38 patients, 25 (66%) showed an increase in cf-PWV post-EVAR, whereas in 13 (34%) patients the cf-PWV decreased, with individual changes ranging from −1.8 to +15.3 m/s. Baseline characteristics were compared between patients who had an increase or a decrease in cf-PWV. Patients with an increase in cf-PWV after EVAR were significantly older (mean age 71.1 vs. 65.5 years; *p* = 0.047), and had higher baseline systolic blood pressure (SBP) (SBP 138 vs. 122 mmHg; *p* = 0.042). No other baseline characteristics differed significantly between the groups. The majority of patients showed increases of 0.5–5.0 m/s, indicating a consistent overall rise in cf-PWV. Two patients exhibited much higher increases, representing clear outliers.

Pre-EVAR, 6 patients (16%) had a cf-PWV above the ESC cut-off value of 12 m/s. After the intervention, 15 patients (39%) exceeded this threshold, indicating a substantial increase in AoS. Five of the preoperative patients with elevated PWV remained above the cut-off postoperatively, while one patient decreased below the threshold.

### 3.2. Subendocardial Viability Ratio

**Figure 4:**
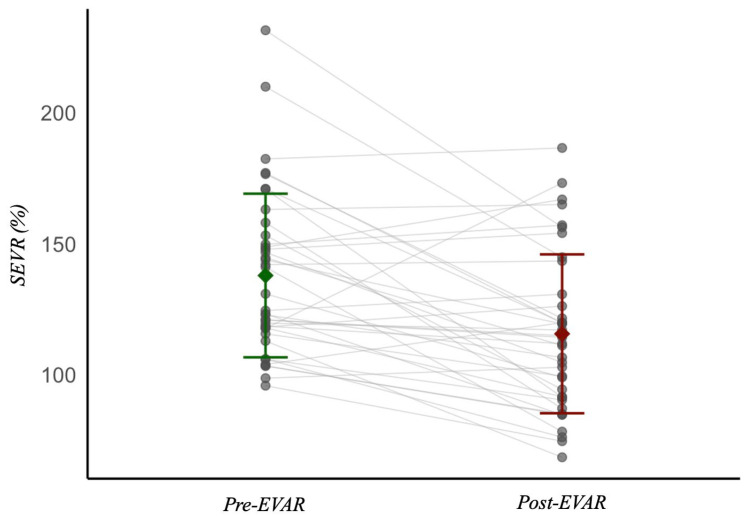
SEVR pre- and post-operatively for EVAR (15.1% decrease, *p* = 0.0058). Each dot represents an individual patient measurement, and lines connect paired pre-EVAR and post-EVAR measurements within the same patient.

SEVR data were normally distributed. Post-EVAR, the mean SEVR significantly decreased from 136.1 ± 5.1% to 115.6 ± 5.1%, a mean reduction of −20.5% (95% CI −34.9 to −6.1; *p* = 0.0058), corresponding to a 15.1% decrease. Figure 4 illustrates the decline in SEVR following EVAR, showing a downward trend across all patients.

Of the 38 patients, 28 (74%) showed a decrease in SEVR, while 10 (26%) exhibited an increase, with changes ranging from −79.5% to +55.5%. Baseline characteristics were compared between patients who had an increase or a decrease in SEVR. The only significant difference in baseline characteristics between the increase and decrease groups was smoking status (*p* = 0.030), with a higher proportion of current/ex-smokers in the SEVR increase group. All other baseline characteristics showed no significant differences. Most patients experienced SEVR changes between −40% and +10%, indicating an overall consistent decrease across the cohort. A few patients showed more extreme increases or decreases, representing outliers.

### 3.3. Graft Length

**Figure 5:**
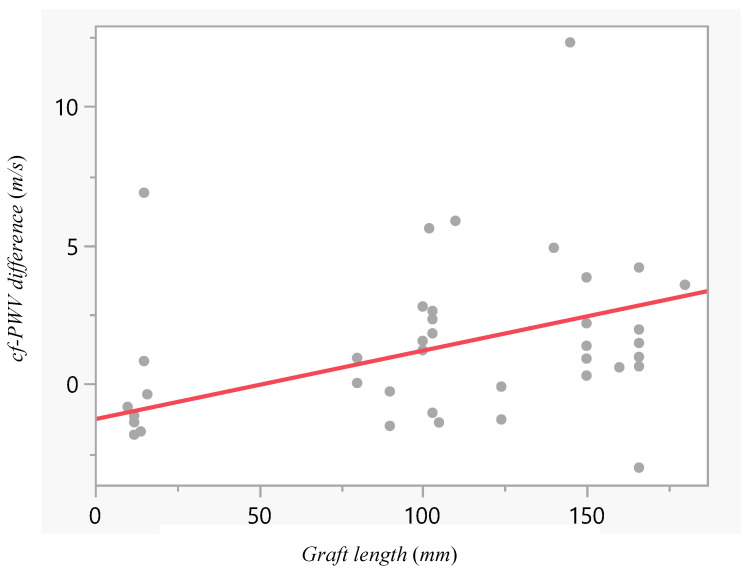
Linear regression of graft length and the postoperative change in cf-PWV. A significant positive correlation was observed (r = 0.41, *p* = 0.029), with a regression coefficient of β = 0.025. Each point represents an individual measurement, and the solid line shows the linear regression fit.

**Figure 6:**
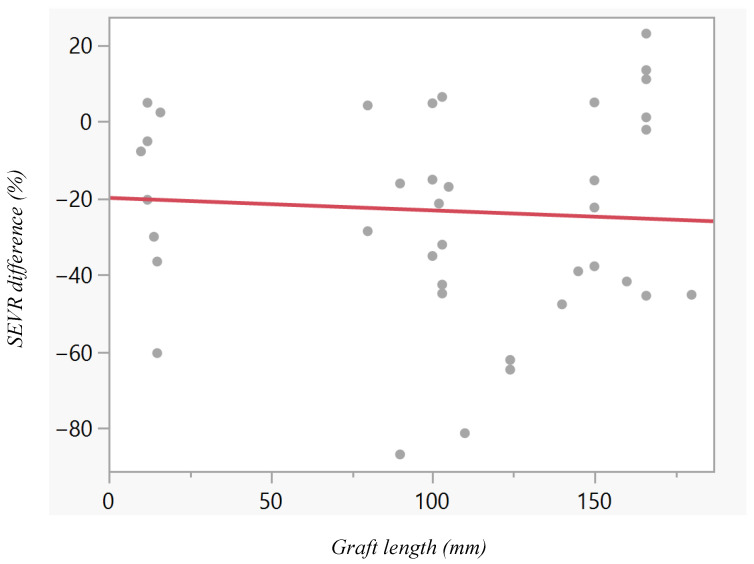
Linear regression of graft length and the postoperative change in SEVR. No significant correlation was found between graft length and SEVR change (r = 0.058, *p* = 0.763), with a regression coefficient of β = −0.033. Each point represents an individual measurement, and the solid line shows the linear regression fit.

Linear regression analysis demonstrated a significant positive correlation between graft length and the change in cf-PWV (r = 0.41; 95%CI 0.045 to 0.672; *p* = 0.029, Figure 5). The regression coefficient was β = 0.0247 ± 0.011, with the regression model expressed as: ΔPWV = −1.28 + 0.0247 × graft length (mm). Relative to the baseline cf-PWV, this coefficient represents a 0.2% increase in cf-PWV per millimetre of graft length. Figure 6 shows the linear regression analysis; despite some variability in individual responses, the upward trend remained consistent across the cohort, underscoring a robust positive relationship between graft length and cf-PWV.

No significant correlation was observed between graft length and the postoperative change in SEVR (r = 0.058; 95% CI −0.30 to 0.40; *p* = 0.763; Figure 6). The regression coefficient was β = −0.033 ± 0.107, with the regression model expressed as: ΔSEVR = −20.03 − 0.033 × graft length (mm).

## 4. Discussion

In this study, EVAR was associated with a significant increase of 10.6% in cf-PWV and a 15.1% reduction in the SEVR, reflecting increased AoS and impaired subendocardial perfusion. Additionally, graft length showed a positive association with the increase in cf-PWV, corresponding to a 0.2% rise in cf-PWV per millimetre of graft length. In contrast, no association was observed between graft length and changes in SEVR. The cohort represents a typical older EVAR population in which both baseline comorbidities and pharmacologic treatment may modulate vascular and cardiac function, providing important context for interpreting post-procedural haemodynamic changes.

**Figure 7:**
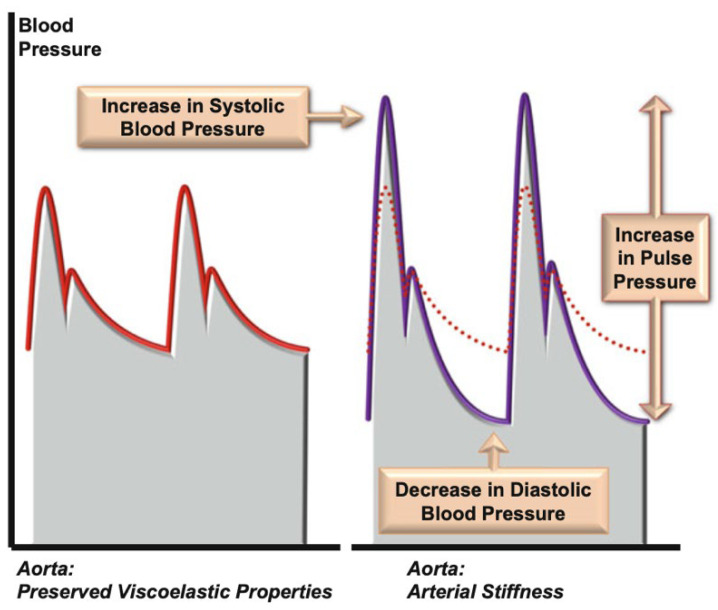
Impact of aortic stiffness on blood pressure. In a native aorta, the viscoelastic properties of the aorta buffer the stroke volume after systolic ejection and create a propulsive effect during diastole. When arterial stiffness occurs, the reduced compliance of the aorta reduces this propulsive effect, leading to an increase in systolic blood pressure, a decrease in diastolic pressure, and an increase in pulse pressure. Reprinted with permission from Springer Nature [3].

The haemodynamic consequences of stent-graft implantation can explain the observed changes in cf-PWV and the SEVR [4,6,26]. Although PWV is influenced by acute haemodynamic conditions, the standardized within-subject design and uncomplicated early postoperative course of the cohort support a predominant contribution of altered aortic wall mechanics to the observed changes. The introduction of a rigid endograft into the compliant native aorta disrupts the normal viscoelastic properties of the vessel, reducing its capacity to expand during systole and recoil during diastole, a phenomenon known as the Windkessel effect. This results in decreased volume storage during systole and greater direct transmission of stroke volume to the periphery. Additionally, the contribution of the aorta to diastolic flow is diminished. This stiffening consequently leads to increased systolic pressure, decreased diastolic pressure, and therefore widened pulse pressure [3] (Figure 7). These alterations accelerate pulse-wave propagation, reflected by increased cf-PWV [3].

These haemodynamic alterations have important clinical implications. EVAR is the preferred treatment for infrarenal abdominal aortic aneurysms, and its widespread use means that post-procedural haemodynamic changes may have substantial population-level implications [16,20]. In our cohort, 15 patients (39%) exceeded the ESC cut-off value of 12 m/s post-EVAR, a threshold recognized as a predictor of increased cardiovascular morbidity and mortality [1,6,22]. A meta-analysis has shown that patients with high PWV have over twice the risk of cardiovascular events (RR 2.26) and death (RR 2.02) compared to those with low PWV [1]. The mechanisms underlying this association can be explained by the haemodynamic consequences of increased aortic stiffness. The rise in systolic blood pressure increases cardiac afterload and myocardial workload, while the reduction in diastolic pressure compromises coronary perfusion, reflected in decreased SEVR [3,4,26]. Thus, the supply of blood to the myocardium decreases, while the heart works harder as afterload increases, possibly leading to left ventricular hypertrophy and an increase in oxygen demand. Moreover, increased pulse pressure may increase the pulsatility of flow to peripheral organs. This higher-pressure wave might promote microvascular damage, particularly in organs that thrive under a consistent blood flow, such as the brain and kidneys [4]. Collectively, these haemodynamic changes indicate that endovascular repair can alter cardiac function, potentially predisposing patients to increased long-term cardiovascular risk, such as impaired coronary perfusion, left ventricular hypertrophy, and ultimately heart failure. Given the high number of EVAR procedures, increases in aortic stiffness could place a considerable proportion of patients at elevated long-term cardiovascular risk.

The observed increase in cf-PWV aligns with previous experimental and clinical studies [13,17,26,27]. Ex vivo porcine studies have reported increases in PWV after stent-graft deployment, with longer graft extensions resulting in larger increases in AoS [10,17,18,27]. Schellinger and colleagues showed that endograft implantation immediately and permanently increased AoS within the stented aortic segment in a porcine ex vivo model [27]. These findings are supported by in vivo human studies, which have reported significant increases of 1.0 m/s in PWV following EVAR [12,13]. Moreover, a recent meta-analysis confirmed significant postoperative increases in PWV after EVAR of 2.97 m/s, highlighting that increases in PWV after EVAR have been documented in previous studies [9]. Together, these data suggest that endovascular stent-grafting consistently leads to increased aortic stiffness, both in experimental models and clinical settings.

A decrease in the SEVR indicates reduced myocardial perfusion and is a parameter to predict the risk of future cardiovascular events and mortality [28,29]. In this study, a significant decrease in the SEVR was observed after EVAR. The results suggest that endovascular aortic procedures may influence myocardial perfusion dynamics, increasing the risk of ischemic events and adversely affecting myocardial function. Previous studies have demonstrated a correlation between increased AoS and decreased SEVR, consistent with the findings of the present study [30,31,32]. Only two other studies have specifically researched the change in the SEVR after endovascular repair, and both showed no statistical difference pre- and post-intervention [33,34]. This may be explained by differences in sample size, as these studies included relatively small cohorts, 20 and 7 patients, respectively.

Graft length and material properties significantly influence postoperative arterial stiffness. Longer stented segments replace a larger proportion of the compliant native aorta, resulting in higher PWV and AoS. Previous data suggest that each additional millimetre of graft length is associated with a 0.54% increase in PWV, consistent with our findings that show a 0.2% increase per mm [35]. Additionally, the mechanical characteristics of the stent graft material play a crucial role in AoS. Grafts composed of stiffer materials tend to increase arterial rigidity more than compliant designs. Stiffer polyester grafts and exoskeletal frameworks are associated with greater PWV increases, whereas polytetrafluoroethylene (PTFE) grafts and endoskeleton designs preserve compliance more effectively [36,37,38]. These differences may be explained by the ability of PTFE and endoskeleton grafts to absorb and transmit pulsatile energy in a manner that more closely resembles the native aortic wall.

From a clinical perspective, the observed increase in AoS following endovascular repair underscores the importance of structured cardiovascular management in the postoperative period. Beyond routine surveillance for graft-related complications, monitoring arterial stiffness and myocardial perfusion parameters should be integrated into follow-up protocols, since these indices provide early markers of adverse haemodynamic adaptation. Postoperative haemodynamic monitoring refers to a structured assessment of arterial stiffness (cf-PWV), myocardial perfusion (SEVR), and blood pressure measurements after stent-graft implantation. Incorporating cf-PWV and SEVR measurements into postoperative care may allow clinicians to identify patients at risk of impaired coronary perfusion or progressive ventricular remodelling before overt clinical deterioration occurs. This allows individualized intervention, including pharmacologic optimization and lifestyle modification. Antihypertensive agents with proven effects on arterial compliance, such as angiotensin-converting enzyme (ACE) inhibitors, angiotensin receptor blockers, and calcium channel blockers, may mitigate the haemodynamic burden imposed by stent-graft implantation [39]. Beta-blockers primarily reduce heart rate and myocardial oxygen demand, but they may also confer indirect benefits by lowering pulsatile load [40]. Furthermore, the choice of therapy should be individualised, considering baseline comorbidities, graft characteristics, and sex-specific vascular responses. Patient selection is critical for endovascular repair. While EVAR is preferred for infrarenal abdominal aortic aneurysms due to its minimally invasive nature, open repair remains an alternative and generally preserves aortic compliance, resulting in less postoperative increase in AoS [34]. Systematic reviews corroborate that PWV increases after EVAR, but not after open repair [9]. Although EVAR has perioperative survival advantages, patients with cardiovascular risk factors, low baseline aortic compliance, or long stent-grafts may be more susceptible to adverse haemodynamic effects. Preoperative assessment of arterial stiffness and myocardial perfusion (cf-PWV, SEVR) may help guide procedure choice and optimize outcomes.

Lifestyle modifications remain a cornerstone of cardiovascular risk reduction. Regular aerobic exercise has been shown to improve endothelial function, reduce arterial stiffness, and enhance myocardial perfusion [41]. Dietary interventions, particularly those rich in omega-3 fatty acids and antioxidants and low in sodium, can further support vascular health [42,43,44]. Smoking cessation and weight optimisation are equally critical, given their established associations with arterial stiffening and impaired coronary flow reserve [45]. Particular attention should be given to high-risk subgroups. Patients with longer grafts, in whom a greater proportion of compliant native aorta is replaced, and postmenopausal women, who may be at higher risk of adverse cardiovascular risks. These groups may benefit from closer haemodynamic monitoring, earlier initiation of compliance-enhancing therapies, and tailored lifestyle counselling.

These preliminary findings provide a foundation for ongoing research. In the current study, we plan to include a larger cohort and follow-up assessments at 6 and 12 months to evaluate the long-term effects of endovascular repair on aortic stiffness. Future studies should investigate the impact of patient-specific characteristics, graft type, the anatomical segment of the aorta involved in the intervention, and the extent of aortic coverage on changes in aortic stiffness. A deeper understanding of these factors may help optimize patient selection and improve outcomes following endovascular repair. Additionally, the scarcity of studies assessing the SEVR underscores the need for larger, prospective studies to clarify the myocardial consequences after endovascular intervention. Furthermore, patient-specific computational modelling may assist in predicting the haemodynamic consequences of stent graft implantation and guide the selection of appropriate graft types for individual patients. Finally, endograft manufacturers should be encouraged to develop next-generation stent grafts that more accurately mimic the viscoelastic properties of the native aorta.

## 5. Limitations

While our study provides valuable insights into the impact of endovascular aortic repair on aortic stiffness, certain limitations should be acknowledged. The sample size was relatively small, which may affect the generalizability of the findings. It should also be noted that the conclusions drawn from this study are based on a relatively small cohort with substantial inter-individual variability in PWV and SEVR, and should therefore be interpreted cautiously as preliminary findings. Additionally, PWV is influenced by acute haemodynamic conditions, including blood pressure and heart rate. Although measurements were performed using a standardized protocol with simultaneous brachial blood pressure assessment and waveform calibration, we did not perform multivariable adjustment for perioperative changes in blood pressure, heart rate, or medication use. Therefore, while the observed increase in PWV is most consistent with altered aortic wall mechanics following stent-graft implantation, particularly given the uncomplicated perioperative course, residual confounding by early postoperative physiology cannot be fully excluded in this preliminary cohort. Furthermore, despite prior validation of the PulsePen^®^, stable pulse wave acquisition could not always be achieved under postoperative conditions, which resulted in the exclusion of a subset of patients due to insufficient waveform quality. Moreover, the stringent exclusion criteria applied to maintain data integrity further reduced the number of eligible participants, potentially leading to sampling bias. Additionally, graft length was quantified based solely on the nominal length of the endograft main body, without accounting for iliac limb extensions. As a result, the total implanted graft length may have been underestimated in cases where limb extensions were used, which could have influenced the observed associations between graft length and changes in cf-PWV and SEVR. Finally, these preliminary findings pertain only to early postoperative changes in PWV and do not include mid- or long-term follow-up; these aspects will be addressed in subsequent phases of the ongoing study. Nevertheless, prior studies have shown that PWV is already elevated shortly after EVAR and remains increased at longer-term follow-up (up to 1 year), with higher early PWV correlating with adverse structural and clinical outcomes, supporting the relevance of acute PWV measurements even in the absence of long-term data in the current cohort [34]. Despite these factors, the consistency of our results suggests a meaningful trend that warrants further investigation.

## 6. Conclusions

Endovascular aortic repair was associated with increased aortic stiffness and reduced subendocardial perfusion. EVAR produced a significant rise in cf-PWV and a reduction in SEVR. These effects were more pronounced in patients with longer grafts. The findings highlight the importance of postoperative haemodynamic monitoring and suggest that patient selection, medical therapy, and future stent-graft design should aim to mitigate these adverse changes. Longer-term studies are warranted to determine whether these early haemodynamic alterations translate into increased cardiovascular morbidity and mortality.

These findings highlight the impact of endovascular aortic repair on aortic stiffness and myocardial perfusion. Our findings serve as a foundation for ongoing research, laying the groundwork for future studies aimed at elucidating the complex relationship between endovascular procedures, AoS, and cardiovascular health. Planned follow-up assessments at 6 and 12 months and analyses considering patient-specific characteristics, graft type, anatomical segment of the aorta, and aortic coverage will help clarify the long-term haemodynamic consequences and optimize patient selection and outcomes following endovascular aortic repair. Ultimately, understanding the dynamic interplay between endovascular repair and arterial biomechanics is crucial for enhancing patient management strategies and reducing the cardiovascular risks linked to these interventions.

## Figures and Tables

**Table 1:** Demographic and clinical characteristics of patients undergoing endovascular aortic repair. * Values are reported as mean ± SD. BMI = Body Mass Index. CKD = Chronic Kidney Disease. COPD = Chronic Obstructive Pulmonary Disease. CABG = Coronary Artery Bypass Grafting. SpO_2_ = Peripheral capillary Oxygen Saturation.

Parameters	EVAR
N	38
Sex	
Male	34 (89%)
Female	4 (11%)
Age	
≥65 y	31 (82%)
Anthropometric characteristics	
BMI (kg/m^2^)	25.5 ± 3.7
Comorbidities	
Hypertension	24 (63%)
Dyslipidaemia	15 (39%)
Diabetes Mellitus	5 (13%)
Atrial Fibrillation	7 (18%)
CKD	1 (3%)
COPD	3 (8%)
Current smoker	8 (21%)
CABG	3 (8%)
Pacemaker	0 (0%)
Medication	
Anti-hypertensives	20 (53%)
β-Blockers	10 (26%)
α-Blockers	3 (8%)
Antiarrhythmics	6 (16%)
Antiplatelet	19 (50%)
Anticoagulants	5 (13%)
Statins	20 (53%)
Proton pump inhibitors	14 (37%)
Vital signs *	
Heart rate	66.91 ± 8.99
SpO_2_ (%)	96.77 ± 1.17
Laboratory values *	
Red Blood Cell Count	4.7 ± 0.6
Haematocrit (%)	41.7 ± 3.5
Haemoglobin (g/dL)	14.2 ± 1.4
Glucose (mg/dL)	107.5 ± 21.6
eGFR (mL/min/1.73 m^2^)	68.2 ± 18.2

**Table 2:** Intra-operative Characteristics of Patients Endovascular Aortic Repair. * Values are reported as mean ± SD. ^†^ These measurements were used to calculate the length of pulse wave propagation, not for surgical purposes.

Parameters	EVAR
N	38
Prosthesis manufacturer	
Medtronic	16 (42%)
Gore	12 (32%)
Terumo	2 (5%)
Endologic	5 (13%)
Cook	1 (3%)
Artivion	1 (3%)
Artivion + Gore	1 (3%)
Graft sizing *	
Graft diameter proximal (mm)	25.5 ± 5.0
Graft diameter distal (mm)	15.8 ± 9.2
Graft length (mm)	101.9 ± 4.6
Right iliac extension diameter (mm)	15.3 ± 1.7
Right iliac extension length (mm)	96.8 ± 57.2
Left iliac extension diameter (mm)	16.2 ± 1.7
Left iliac extension length (mm)	93.5 ± 56.7
Anatomic segment length *^,†^	
Carotid-femoral length (mm)	610.7 ± 45.9
Carotid-sternal length (mm)	115.3 ± 20.7
Sternal-femoral length (mm)	540.1 ± 48.6
Anatomical segment diameter *	
Aortic diameter (mm)	51.9 ± 11.4
Iliac diameter (mm)	30.5 ± 6.5
Aortic neck diameter (mm)	50.8 ± 10.3
Length of stay (days) *	5.0 ± 2.2

## Data Availability

All data supporting the findings of this study will be made available by the corresponding author upon reasonable request.

## References

[1] Vlachopoulos C., Aznaouridis K., Stefanadis C. (2010). Prediction of cardiovascular events and all-cause mortality with arterial stiffness: A systematic review and meta-analysis. J. Am. Coll. Cardiol..

[2] Chirinos J.A., Segers P., Hughes T., Townsend R. (2019). Large-Artery Stiffness in Health and Disease: JACC State-of-the-Art Review. J. Am. Coll. Cardiol..

[3] Salvi P. (2012). Pulse Waves.

[4] Palombo C., Kozakova M. (2016). Arterial stiffness, atherosclerosis and cardiovascular risk: Pathophysiologic mechanisms and emerging clinical indications. Vasc. Pharmacol..

[5] Mattace-Raso F.U., van der Cammen T.J., Hofman A., van Popele N.M., Bos M.L., Schalekamp M.A., Asmar R., Reneman R.S., Hoeks A.P., Breteler M.M. (2006). Arterial stiffness and risk of coronary heart disease and stroke: The Rotterdam Study. Circulation.

[6] Mitchell G.F., Hwang S.J., Vasan R.S., Larson M.G., Pencina M.J., Hamburg N.M., Vita J.A., Levy D., Benjamin E.J. (2010). Arterial stiffness and cardiovascular events: The Framingham Heart Study. Circulation.

[7] Ben-Shlomo Y., Spears M., Boustred C., May M., Anderson S.G., Benjamin E.J., Boutouyrie P., Cameron J., Chen C.H., Cruickshank J.K. (2014). Aortic pulse wave velocity improves cardiovascular event prediction: An individual participant meta-analysis of prospective observational data from 17,635 subjects. J. Am. Coll. Cardiol..

[8] Laurent S., Boutouyrie P., Asmar R., Gautier I., Laloux B., Guize L., Ducimetiere P., Benetos A. (2001). Aortic stiffness is an independent predictor of all-cause and cardiovascular mortality in hypertensive patients. Hypertension.

[9] Bissacco D., Conti M., Domanin M., Bianchi D., Scudeller L., Mandigers T.J., Allievi S., Auricchio F., Trimarchi S. (2022). Modifications in Aortic Stiffness After Endovascular or Open Aortic Repair: A Systematic Review and Meta-Analysis. Eur. J. Vasc. Endovasc. Surg..

[10] Mandigers T.J., Conti M., Allievi S., Dedola F., Bissacco D., Bianchi D., Marconi S., Domanin M., Van Herwaarden J.A., Auricchio F. (2023). Comparison of Two Generations of Thoracic Aortic Stent Grafts and Their Impact on Aortic Stiffness in an Ex Vivo Porcine Model. EJVES Vasc. Forum.

[11] van Herwaarden J.A., Muhs B.E., Vincken K.L., van Prehn J., Teutelink A., Bartels L.W., Moll F.L., Verhagen H.J. (2006). Aortic compliance following EVAR and the influence of different endografts: Determination using dynamic MRA. J. Endovasc. Ther..

[12] Kadoglou N.P., Moulakakis K.G., Papadakis I., Ikonomidis I., Alepaki M., Lekakis J., Liapis C.D. (2012). Changes in aortic pulse wave velocity of patients undergoing endovascular repair of abdominal aortic aneurysms. J. Endovasc. Ther..

[13] Gray C., Goodman P., Badger S.A., O’Malley M.K., O’Donohoe M.K., McDonnell C.O. (2016). Endovascular Aneurysm Repair Increases Aortic Arterial Stiffness When Compared to Open Repair of Abdominal Aortic Aneurysms. Vasc. Endovasc. Surg..

[14] Lederle F.A., Kyriakides T.C., Stroupe K.T., Freischlag J.A., Padberg F.T., Matsumura J.S., Huo Z., Johnson G.R. (2019). Open versus Endovascular Repair of Abdominal Aortic Aneurysm. N. Engl. J. Med..

[15] Riambau V., Böckler D., Brunkwall J., Cao P., Chiesa R., Coppi G., Czerny M., Fraedrich G., Haulon S., Jacobs M.J. (2017). Editor’s Choice—Management of Descending Thoracic Aorta Diseases: Clinical Practice Guidelines of the European Society for Vascular Surgery (ESVS). Eur. J. Vasc. Endovasc. Surg..

[16] Czerny M., Grabenwöger M., Berger T., Aboyans V., Della Corte A., Chen E.P., Desai N.D., Dumfarth J., Elefteriades J.A., Etz C.D. (2024). EACTS/STS Guidelines for Diagnosing and Treating Acute and Chronic Syndromes of the Aortic Organ. Ann. Thorac. Surg..

[17] de Beaufort H.W.L., Coda M., Conti M., van Bakel T.M.J., Nauta F.J.H., Lanzarone E., Moll F.L., van Herwaarden J.A., Auricchio F., Trimarchi S. (2017). Changes in aortic pulse wave velocity of four thoracic aortic stent grafts in an ex vivo porcine model. PLoS ONE.

[18] de Beaufort H.W.L., Conti M., Kamman A.V., Nauta F.J.H., Lanzarone E., Moll F.L., van Herwaarden J.A., Auricchio F., Trimarchi S. (2017). Stent-Graft Deployment Increases Aortic Stiffness in an Ex Vivo Porcine Model. Ann. Vasc. Surg..

[19] Bissacco D., Grignaffini C., Romagnoli S., Gherbesi E., Domanin M., Casana R., Salvi P., Parati G., Gallo D., Carugo S. (2024). A study protocol for evaluating aortic stiffness modifications in patients treated with endovascular aortic repair. Minerva Cardiol. Angiol..

[20] Wanhainen A., Verzini F., Van Herzeele I., Allaire E., Bown M., Cohnert T., Dick F., van Herwaarden J., Karkos C., Koelemay M. (2019). Editor’s Choice—European Society for Vascular Surgery (ESVS) 2019 Clinical Practice Guidelines on the Management of Abdominal Aorto-iliac Artery Aneurysms. Eur. J. Vasc. Endovasc. Surg..

[21] Upchurch G.R., Escobar G.A., Azizzadeh A., Beck A.W., Conrad M.F., Matsumura J.S., Murad M.H., Perry R.J., Singh M.J., Veeraswamy R.K. (2021). Society for Vascular Surgery clinical practice guidelines of thoracic endovascular aortic repair for descending thoracic aortic aneurysms. J. Vasc. Surg..

[22] (2010). The Reference Values for Arterial Stiffness’ Collaboration. Determinants of pulse wave velocity in healthy people and in the presence of cardiovascular risk factors: ‘establishing normal and reference values’. Eur. Heart J..

[23] Salvi P., Lio G., Labat C., Ricci E., Pannier B., Benetos A. (2004). Validation of a new non-invasive portable tonometer for determining arterial pressure wave and pulse wave velocity: The PulsePen device. J. Hypertens..

[24] Salvi P., Grillo A., Gautier S., Labat C., Salvi L., Valbusa F., Baldi C., Rovina M., Simon G., Gao L. (2024). Myocardial oxygen supply and demand imbalance predicts mortality in older nursing home residents: The PARTAGE study. J. Am. Geriatr. Soc..

[25] Schott A., Kluttig A., Mikolajczyk R., Großkopf A., Greiser K.H., Werdan K., Sedding D., Nuding S. (2024). Association of subendocardial viability ratio and mortality in the elderly population: Results from the CARdiovascular disease, Living and Ageing in Halle study. J. Hypertens..

[26] Takeda Y., Sakata Y., Ohtani T., Tamaki S., Omori Y., Tsukamoto Y., Aizawa Y., Shimamura K., Shirakawa Y., Kuratani T. (2014). Endovascular aortic repair increases vascular stiffness and alters cardiac structure and function. Circ. J..

[27] Schellinger I.N., Naumann J., Hoffmann A., Barnard S.J., Düsing S., Wagenhäuser M.U., Haunschild J., Scheinert D., Hasenfuß G., Etz C.D. (2025). Abdominal Aortic Endograft Implantation Immediately Induces Vascular Stiffness Gradients That May Promote Adverse Aortic Neck Dilatation: Results of A Porcine Ex Vivo Study. J. Endovasc. Ther..

[28] Xie H., Fan F., Gao L., Zhang X., Jia J., Gong Y., Zhang Y. (2025). Association between subendocardial viability ratio and cardiovascular events: A cohort study in a Chinese community-based population. BMJ Open.

[29] Xie H., Gao L., Fan F., Gong Y., Zhang Y. (2024). Research Progress and Clinical Value of Subendocardial Viability Ratio. J. Am. Heart Assoc..

[30] Hashimoto J., Ito S. (2017). Central diastolic pressure decay mediates the relationship between aortic stiffness and myocardial viability: Potential implications for aortosclerosis-induced myocardial ischemia. J. Hypertens..

[31] Scandale G., Dimitrov G., Recchia M., Carzaniga G., Minola M., Perilli E., Carotta M., Catalano M. (2018). Arterial stiffness and subendocardial viability ratio in patients with peripheral arterial disease. J. Clin. Hypertens..

[32] Anyfanti P., Gkaliagkousi E., Triantafyllou A., Dipla K., Zarifis H., Arseniou P., Lazaridis A., Douma S. (2019). Noninvasive Assessment of Myocardial Perfusion in Different Blood Pressure Phenotypes and Its Association with Arterial Stiffness Indices. Am. J. Hypertens..

[33] Moloney M.A., McHugh S., O’Donnell D.H., Casey R.G., Kavanagh E.G., Grace P.A., Fitzgerald P., Bouchier-Hayes D.J. (2011). Comparison of arterial stiffness and microcirculatory changes following abdominal aortic aneurysm grafting. Ir. J. Med. Sci..

[34] Holewijn S., Vermeulen J.J.M., van Helvert M., van de Velde L., Reijnen M. (2021). Changes in Noninvasive Arterial Stiffness and Central Blood Pressure After Endovascular Abdominal Aneurysm Repair. J. Endovasc. Ther..

[35] Abatzis-Papadopoulos M., Tigkiropoulos K., Nikas S., Antza C., Alexou C., Lazaridi A.M., Stavridis K., Kotsis V., Lazaridis I., Saratzis N. (2025). Treatment Length and External Iliac Artery Extension Are Associated with Increased Aortic Stiffness After Endovascular Aortic Repair: A Prospective, Monocentric, Single-Arm Study. Biomedicines.

[36] Kadoglou N.P., Moulakakis K.G., Papadakis I., Ikonomidis I., Alepaki M., Spathis A., Karakitsos P., Lekakis J., Liapis C.D. (2014). Differential effects of stent-graft fabrics on arterial stiffness in patients undergoing endovascular aneurysm repair. J. Endovasc. Ther..

[37] Sultan S., Acharya Y., Soliman O., Parodi J.C., Hynes N. (2022). TEVAR and EVAR, the unknown knowns of the cardiovascular hemodynamics; and the immediate and long-term consequences of fabric material on major adverse clinical outcome. Front. Surg..

[38] Hori D., Yuri K., Kusadokoro S., Shimizu T., Kimura N., Yamaguchi A. (2020). Effect of endoprostheses on pulse wave velocity and its long-term outcomes after thoracic endovascular aortic repair. Gen. Thorac. Cardiovasc. Surg..

[39] Safar M.E., Asmar R., Benetos A., Blacher J., Boutouyrie P., Lacolley P., Laurent S., London G., Pannier B., Protogerou A. (2018). Interaction Between Hypertension and Arterial Stiffness. Hypertension.

[40] Safar M.E., Blacher J., Mourad J.J., London G.M. (2000). Stiffness of carotid artery wall material and blood pressure in humans: Application to antihypertensive therapy and stroke prevention. Stroke.

[41] Liu H., Shivgulam M.E., Schwartz B.D., Kimmerly D.S., O’Brien M.W. (2023). Impact of exercise training on pulse wave velocity in healthy and clinical populations: A systematic review of systematic reviews. Am. J. Physiol. Heart Circ. Physiol..

[42] D’Elia L., Galletti F., La Fata E., Sabino P., Strazzullo P. (2018). Effect of dietary sodium restriction on arterial stiffness: Systematic review and meta-analysis of the randomized controlled trials. J. Hypertens..

[43] Stanek A., Grygiel-Górniak B., Brożyna-Tkaczyk K., Myśliński W., Cholewka A., Zolghadri S. (2023). The Influence of Dietary Interventions on Arterial Stiffness in Overweight and Obese Subjects. Nutrients.

[44] Monahan K.D., Feehan R.P., Blaha C., McLaughlin D.J. (2015). Effect of omega-3 polyunsaturated fatty acid supplementation on central arterial stiffness and arterial wave reflections in young and older healthy adults. Physiol. Rep..

[45] Czernin J., Sun K., Brunken R., Böttcher M., Phelps M., Schelbert H. (1995). Effect of acute and long-term smoking on myocardial blood flow and flow reserve. Circulation.

